# Influence of anxiety sensitivity on the observe facet of the five facet mindfulness questionnaire: differential item functioning in a clinical population

**DOI:** 10.1186/s12888-025-06488-x

**Published:** 2025-04-01

**Authors:** Danielle Moskow Diamond, Joshua Curtiss, Joseph K. Carpenter, Masaya Ito, Stefan G. Hofmann

**Affiliations:** 1 Montefiore Medical Center/Albert Einstein College of Medicine, Bronx, NY USA; 2 Bouvé College of Health Sciences, Northeastern University, Boston, MA USA; 3 National Center for PTSD, VA Boston Healthcare System, Boston, MA USA; 4 Boston University School of Medicine, Boston, MA USA; 5 National Center of Neurology and Psychiatry, Tokyo, Japan; 6 Philipps University Marburg, Marburg, Germany

**Keywords:** Anxiety sensitivity, Panic disorder, Mindfulness, Observe, Internal, External, Cognitive

## Abstract

**Background:**

Many individuals who engage in mindfulness experience decreased anxiety. Yet some individuals, particularly those with panic disorder (PD) or elevated anxiety sensitivity (AS), note heightened anxiety when observing particular sensations. The Five Facet Mindfulness Questionnaire (FFMQ) is one of the most widely utilized mindfulness questionnaires. However, its Observe facet has shown variability in the literature. This study explored a transdiagnostic approach to determine whether specific aspects of the Observe facet of the FFMQ differ in individuals with elevated anxiety sensitivity (AS).

**Methods:**

We examined a clinical sample of 1521 Japanese individuals who completed the FFMQ and Anxiety Sensitivity Index 3 (ASI-3). A multiple indicator multiple cause (MIMIC) model approach was adopted utilizing latent variables to examine differential item functioning (DIF) of the Observe facet of the FFMQ, based on PD and/or AS diagnosis. This process was repeated to examine the relationship between the Anxiety Sensitivity Index-3 (ASI-3) subscales and particular items of the Observe facet.

**Results:**

The measurement model revealed good acceptability of a one-factor solution of the Observe facet (χ^2^ (20) = 208.73, *p* < 0.001). PD significantly predicted Observe (B = 0.09, R^2^ = 0.06, *p* = 0.008) and AS significantly predicted Observe (B = 0.02, R^2^ = 0.18, *p* < 0.001). Increased AS was associated with greater scores on observing internal items and lower scores on observing external items. When PD and AS were analyzed simultaneously, only AS remained significant. The cognitive subscale showed the same pattern of results as the total ASI-3 subscale.

**Conclusions:**

The findings of this study reveal that AS, particularly cognitive AS, may modulate the mindfulness experience. The study reveals the importance of understanding where an individual observes and its results may be beneficial for tailoring mindfulness interventions for individuals.

**Supplementary Information:**

The online version contains supplementary material available at 10.1186/s12888-025-06488-x.

## Background

Mindfulness techniques have become pervasive in the field of mental health. Many individuals engage in meditation as a mindfulness practice; however, it is important to note that meditation is only one type of mindfulness. Jon Kabat-Zinn, the pioneer of Western mindfulness, defined mindfulness as “awareness that arises through paying attention, on purpose, in the present moment, non-judgmentally” [1]. Mindfulness, in many ways, is the anthesis of anxiety, as anxiety typically involves future-oriented worries. Mindfulness-based programs (MBPs) have been developed as an effective treatment for stress, depression and anxiety. As opposed to anxious thoughts about the future or rumination about the past, MBPs emphasize increasing awareness toward present moment sensations and experiences, while maintaining a nonjudgmental attitude [2]. As such, research has found that mindfulness is a prominent protective factor for reducing psychological distress [3]. The results of a meta-analysis by Hofmann and colleagues [4] emphasize this finding, as anxiety and mood symptoms were found to significantly improve across 39 studies that utilized mindfulness-based therapy.

Given the plethora of evidence that mindfulness can be beneficial for anxiety, it remains unclear why some individuals struggle with mindfulness. For example, some individuals find that focusing on their breath can feel uncomfortable or even increase anxiety. This is often true for those with panic disorder (PD), as individuals with PD are highly in tune with bodily sensations, frequently observing their internal symptoms and finding these symptoms aversive [5]. Fear of bodily sensations is not just unique to PD, however. Research has found that individuals with elevated anxiety sensitivity (AS) more generally are likely to notice increased anxiety and feel more anxious when observing their physical sensations [6]. AS is the belief that anxiety symptoms are harmful, and AS varies across anxiety and related disorders. As such, individuals who are more sensitive to their anxiety may have increased difficulty engaging in certain mindfulness interventions, such as techniques that involve observing internal sensations. However, several types of MBPs have shown promise for improving AS. In a recent study, Khalili and colleagues [7] studied the effects of mindfulness-based cognitive therapy (MBCT) on AS in patients with multiple sclerosis. The researchers found that MBCT helped reduce AS in these patients. Another recent study by Xie and colleagues [8] assigned participants to either an eight-week mindfulness intervention or a control group waitlist. The results suggested that AS may be a potential mechanism for the impact of mindfulness training on anxiety and depression.

In line with moving towards individualized treatment, it is important to have a more robust understanding of who may benefit from which mindfulness interventions and why. The first step in this process is to better understand our assessment of mindfulness. Several studies have investigated the incremental validity of each facet of the Five Facet Mindfulness Questionnaire (FFMQ) [9]- one of the most widely utilized mindfulness questionnaires- in predicting anxiety, depression and stress [10, 11]. The facets of the FFMQ include Act with Awareness, Describe, Observe, Non-judge and Non-react. The Observe facet was the only factor not found to be a component of Baer and colleagues’ [9] overall mindfulness construct, and this finding was replicated in clinical samples [12]. In fact, Brown and colleagues [13] found that those who scored highly on the Observe facet experienced increased stress and anxiety, which is in line with research noting that frequent internal observation is often associated with increased anxiety in PD and those with higher AS. Diehl and colleagues [14] recently examined the underlying structure of the FFMQ and the relations between the facets and dimensions of psychopathology. The authors noted that all facets except Observe were negatively associated with internalizing psychopathology, providing further and current evidence of differing results with the Observe facet. As observing may increase anxiety for some individuals, this area needs to be better understood in order to recommend specific MBPs to individuals [15].

Previous studies have investigated the relationship between PD and observing and have found that those with PD tend to score higher on the Observe facet of the FFMQ compared to individuals with other anxiety and related disorders [16]. Interpretation of these relationships, however, is complicated by the fact that higher observing scores do not necessarily correlate with higher overall mindfulness scores, as discussed earlier. Another challenge with these findings is the Observe facet of the FFMQ does not differentiate between where one observes- i.e. internally (on physical sensations/thoughts/emotions) or externally (on sights/smells/sounds around them). Relatedly, Baer and colleagues [17] developed the Kentucky Inventory of Mindfulness Skills (KIMS) [18], which consists of Observe, Describe and Act with awareness facets. Baer and colleagues [17] found that those with higher AS had a heightened tendency to observe thoughts, sensations and emotions. The Observe facet was found to tap into a form of hypervigilance to threatening interpersonal experiences in those who were fearful of their psychological and physical sensations. Neuroticism was negatively correlated with all facets except Observe. Waszczuk and colleagues [18] also investigated the relationship between trait mindfulness and AS and described AS as a cognitive bias that may impact the association between mindfulness and anxiety or depression. It would follow that certain types of increased observation may correlate with increased anxiety.

An interesting finding noted by Baer and colleagues across several studies [17, 19] was that higher scores on the Observe facet were associated with stronger psychological adjustment among meditating samples, whereas the relationship in non-meditating individuals was either nonsignificant or in the opposite direction. Thus, it appears that meditation experience may moderate the relationship between observing and AS. A recent study by Moskow Diamond et al. [20] explored whether some facets of mindfulness changed more than others across 12 weeks of kundalini yoga, CBT or a stress education control group in individuals with generalized anxiety disorder. Across all treatments, the Non-judge, Act with Awareness and Non-react facets significantly increased, while the Describe and Observe facets did not. Therefore, meditation may serve a unique function that other types of MBPs do not.

Given the many potential benefits of mindfulness as treatment for anxiety, it is essential for researchers and clinicians to better understand who may benefit from which mindfulness skills and why, so that we can work towards disseminating and implementing MBPs as effective treatment for individuals. Given that the FFMQ is one of the commonly used mindfulness scales [21] and important gaps in this scale exist, it is crucial to investigate the aspects of this scale that are less understood. There is increasingly significant evidence that mindfulness is not only multifaceted, but also certain facets of the FFMQ, such as Observe, appear to operate differently for different people [18].

Most research on this topic has focused on the relationship between PD and increased observation, due to the notion that those who focus internally may find MBPs, such as meditation, to be aversive. This study aimed to better understand the relationship between AS and observation across a clinical, transdiagnostic sample. We believe the Observe facet of the FFMQ is multifaceted, and it may be important to note more precisely what people tend to observe. In this way, we can better determine which types of mindfulness interventions may work best for which individuals. Finally, the FFMQ in the Japanese population has not been as thoroughly explored as in the American population. Thus, studying mindfulness in a Japanese population can improve cross-cultural knowledge of mindfulness more broadly.

### Study aims

**Fig. 1:**
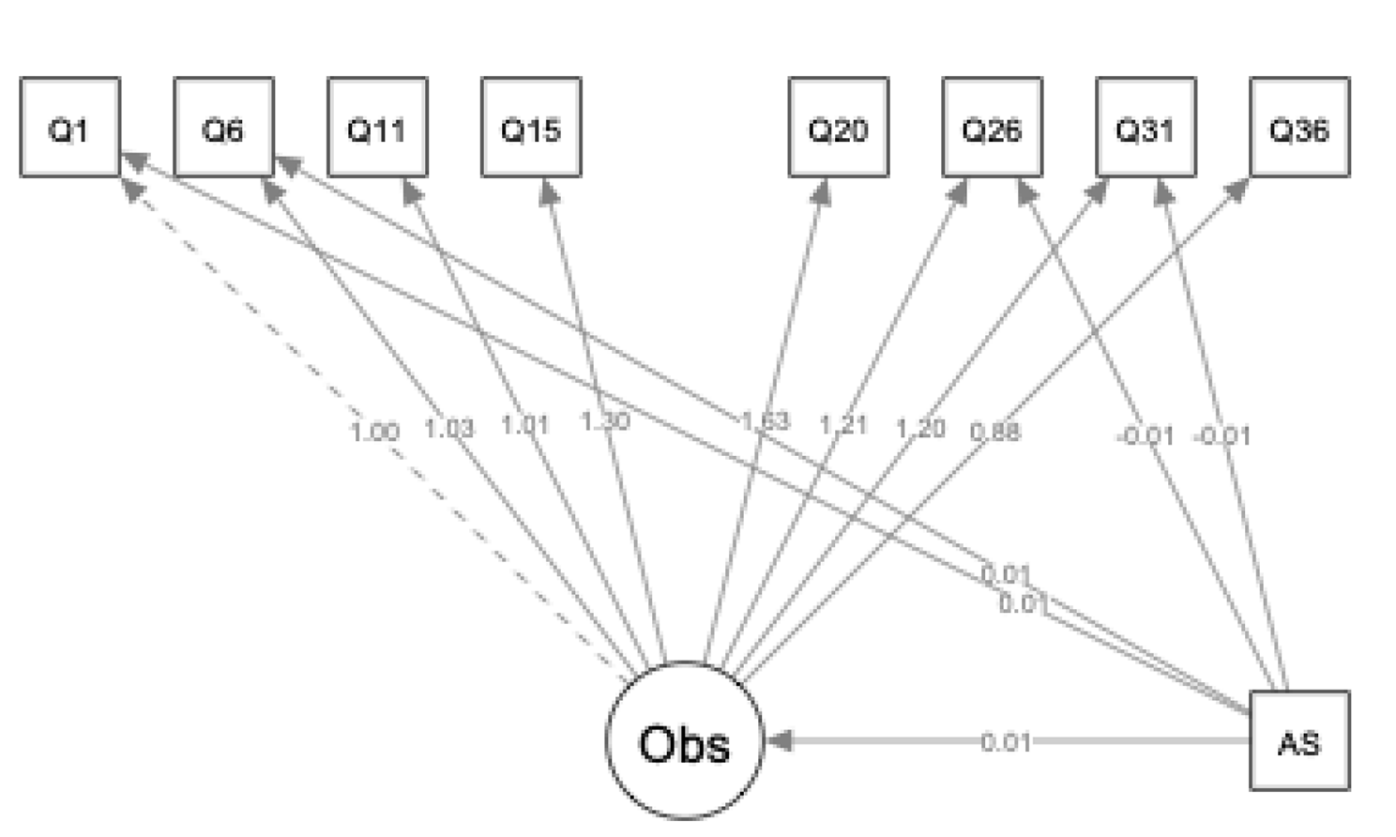
Mimic Model with AS Total Covariate Note: Dashed lines denote loadings fixed to one to scale the latent variable, whereas solid lines denote loadings that are freely estimated. This figure was created using R package semPlot With respect to item content: Q1: “When I’m walking, I deliberately notice the sensations of my body moving;” Q6: “When I take a shower or bath, I stay alert to the sensations of water on my body;” Q11: “I notice how foods and drinks affect my thoughts, bodily sensations, and emotions;” Q15: “I pay attention to sensations, such as the wind in my hair or sun on my face;” Q20: “I pay attention to sounds, such as clocks ticking, birds chirping, or cars passing;” Q26: “I notice the smells and aromas of things;” Q31: “I notice visual elements in art or nature, such as colors, shapes, textures, or patterns of light and shadow;” Q36: “I pay attention to how my emotions affect my thoughts and behavior.”

The goal of this study was to examine the relationship between PD, AS and internal/external items on the Observe facet of the FFMQ. Our first aim was to examine the Observe facet as a single latent variable across a clinical population, and we hypothesized this would reveal a single factor solution, consistent with prior research (See Fig. 1 for full item description). The second aim was to investigate the relation between PD and specific items of the Observe facet using MIMIC modeling to examine differential item functioning (DIF). We hypothesized those with PD would endorse higher levels of Observe overall, as well as higher internal focus and lower external focus, compared to those without PD. The third aim was to investigate the relation between varying levels of AS and specific items of the Observe facet. We hypothesized that individuals who are more sensitive to their anxiety would observe more overall compared to those lower in AS. We predicted individuals with elevated AS would score higher on items measuring internal focus and lower on items measuring external focus. The final aim of the study was to explore the relationship between the ASI-3 subscales and items of the Observe facet that were either more internally or externally focused. We hypothesized that the physical concern subscale of the ASI-3 would be related more to internal focus and less to external focus, compared to the cognitive or social concern subscales.

## Methods

### Participants and procedures

Participants (*n* = 1521) ages 18 years and older were recruited from panelists who were registered at Macromill Incorporation, which is a large internet marketing research company in Japan. The Institutional Review Board at the National Center of Neurology and Psychiatry approved the ethical and scientific validity of this study (approval number: A2013-022). Out of 1,095,443 individuals who are registered panelists, 389,265 individuals are registered as “disease panelists” [22]. Individuals registered as disease panelists reported a current diagnosis of a clinical disorder that had been previously diagnosed by a medical practitioner. For instance, to self-report PD, participants were asked “Are you currently diagnosed as having panic disorder and being treated for the problem in a medical setting?” Similar questions were asked for other diagnoses as well [23]. We based our population for this study on the sample from Curtiss and colleagues [22], who randomly extracted a sample of 2,830 participants from this panelist pool based on gender, age, and living area. All participants signed informed consent and completed questionnaires online.

Participants in this sample reported the following diagnoses: PD (*n* = 198), social anxiety disorder (SAD; *n* = 116), obsessive compulsive disorder (OCD; *n* = 66), major depressive disorder (MDD; *n* = 406), comorbid MDD with anxiety and related disorder (*n* = 636) or comorbid anxiety and related disorders (*n* = 99). The mean age in this sample was 42.42 (*SD =* 9.49), 51% were female and all participants identified as Japanese (see Table 1).

**Table 1:** Demographic and clinical descriptive information

Diagnosis	Male (*N*)	Female (*N*)		Japanese (*N*)	
MDD only	239	167		406	
PD only	84	114		198	
SAD only	43	73		116	
OCD only	29	37	66		
Comorbid MDD & any anxiety disorder or OCD	310	326	636		
Comorbid anxiety disorder or OCD	41	58	99		
Age	Mean (years)	SD			
	42.42	9.49			

Note. ^a^MDD = major depressive disorder; PD = panic disorder; SAD = social anxiety disorder; OCD = obsessive-compulsive disorder

### Measures

#### Five Facet Mindfulness Questionnaire (FFMQ) [9]

This self-report questionnaire is comprised of 39 total questions, made up of five subscales. Responses are rated on a 5-point Likert scale, with scores ranging from 0 (“Never or very rarely true”) to 5 (“Very often or always true”). The Observe facet is comprised of 8 questions (1, 6, 11, 15, 20, 26, 31 and 36, see Fig. 1). The sections are summed for total scores. This scale has been validated in a Japanese sample and has exhibited good psychometric properties [24]. Several studies have investigated the incremental validity of individual facets of the FFMQ in predicting anxiety [10]. All facets of the FFMQ have shown adequate-to-good internal consistency, ranging from 0.72 to 0.92 [19].

#### Anxiety Sensitivity Index-3 (ASI-3)[25]

This 18-item self-report questionnaire was developed from the original ASI [26]. The scale measures the extent to which an individual is worried about negative consequences occurring from their anxiety-related symptoms. The ASI-3 is comprised of three subscales (cognitive, physical and social concerns), each consisting of six items. Participants rate responses on a 5-point Likert scale, with possible scores ranging from 0 (“Very little”) to 4 (“Very much”). Responses are summed for a total overall score. The ASI-3 has shown valid assessment of AS, as well as acceptable to good internal consistency [27]. Acceptable reliability has been shown in each of the three subscales of the ASI-3, with alpha values ranging from 0.73 to 0.91 [25]. This scale was translated to Japanese to be used in this study.

### Statistical analyses

In the first set of analyses, individuals diagnosed with PD were compared to individuals without PD. In the following analyses, all individuals with clinical diagnoses were compared based on varying levels of AS. To examine DIF of the Observe facet of the FFMQ, a multiple indicator multiple cause (MIMIC) model approach was adopted utilizing latent variables. Consistent with the framework posited by Brown [28], an iterative model-building approach was pursued. First, a measurement model of the latent variable of the Observe facet was evaluated. Second, the AS covariate was regressed onto the latent variable and all indicators, yet all pathway coefficients from the covariate to the indicators were fixed to zero. Modification indices were inspected to determine whether there were any salient instances of local model misspecification (i.e. whether freely estimating the regression parameter would substantially improve model fit, as indicated by a modification index exceeding 3.84 and substantive justification). Third, a new latent variable model was specified permitting the covariate to freely predict the latent variable and the indicators that exhibited the largest modification indices in the prior model. After estimation of the MIMIC model, modification indices were reinspected to inform whether any other regression parameters from the covariate to the indicators should be freed. This process was continued until the modification indices and substantive justification revealed no further instances of poor fit. Then, this process was repeated for individual AS subscales and PD as a covariate.

We assessed model fit using four fit indices. The chi-square statistic (χ^2^) can be construed such that smaller values correspond to better fit. As this fit index is especially sensitive to sample size and is overly stringent, three additional fit indices were examined. The Non-Normed Fit Index (NNFI) and the Comparative Fit Index (CFI) were utilized as they exact a penalty for adding parameters, which is not the case with the laxer Normed Fit Index (NFI). The Root Mean Square Error of Approximation (RMSEA) is a measure based on the non-centrality parameter. NNFI and CFI values approaching 0.90 and 0.95 indicate acceptable and good model fit, respectively. RMSEA values between 0.08 and 0.10 indicate marginal fit, values < 0.08 indicate reasonable model fit and values < 0.05 indicate close fit [29, 30]. Modification indices were examined to determine the presence of local areas of model strain. All latent variable analyses were conducted using the Lavaan package, using unweighted least squares with robust standard errors and a mean- and variance-adjusted test statistic, given this estimation approach performs well under violations of normality that are prone to occur with Likert item data [28, 31]. Moreover, post-hoc power analyses were conducted using the RMSEA criterion and an alpha of 0.05 to detect the degree of discrepancy between the model implied and model observed covariance matrices, which provides a general effect size measure commonly considered in structural equation modeling [32].

## Results

Means and standard deviations of the FFMQ and ASI subscales are presented in Table 2.

**Table 2:** Means and SD of FFMQ and ASI subscales

Group	FFMQ Observe	FFMQ Non-react	FFMQ Non-judge	FFMQ Describe	FFMQ Act Aware	ASI Physical	ASI Cog	ASI Social
No PD	20.85 (5.89)	17.14 (4.80)	24.734 (6.47)	20.84 (6.37)	25.89 (6.32)	7.23 (6.28)	8.03 (6.71)	9.6 (6.33)
PD	21.67 (6.297)	17.29 (4.88)	24.58 (6.67)	21.79 (6.28)	26.07 (6.42)	11.011 (7.06)	9.17 (6.99)	10.69 (6.29)

### Measurement model

Results of the measurement model revealed the acceptability of a one-factor solution of the Observe facet. The chi-square statistic was significant (*χ*^2^ (20) = 208.73, *p* < 0.001) and the other indices indicated good global fit: CFI = 0.98, NNFI = 0.97, RMSEA = 0.079 (90% CI: 0.069 to 0.089; see Table 3).

**Table 3:** Results from MIMIC model

	χ^2^	df	CFI	NNFI	RMSEA (90% CI)
Observe Measurement Model	208.73***	20	0.98	0.97	0.079 (0.069, 0.089)
PD to Observe Model	259.98***	27	0.98	0.97	0.072 (0.063, 0.080)
AS to Observe Model	261.16***	27	0.99	0.99	0.076 (0.067, 0.084)
AS MIMIC Model	66.43***	36	0.99	0.99	0.035 (0.026, 0.045)
AS Subscales to Observe Model	208.35***	41	0.98	0.98	0.052 (0.045, 0.059)
AS Subscales MIMIC Model	58.77***	29	0.99	0.99	0.026 (0.016, 0.036)

Note.^b^MIMIC = multiple indicator multiple cause; PD = panic disorder AS = anxiety sensitivity; ^c^*p < 0.05; **p < 0.01; ***p < 0.001

All factor loadings were positive and significant, with unstandardized coefficients ranging from 0.87 to 1.36. Given this consistency with prior literature [9, 12], this specification of the one-factor solution was retained for subsequent analyses.

### Panic disorder

A MIMIC model was pursued to determine whether PD accounts for DIF in indicators of the latent variable of Observe. A measurement model with PD as a covariate was specified wherein the pathways of PD predicting the item indicators were fixed to zero, yet the pathway to Observe was freely estimated. This model was associated with global fit indices that were reasonable to good fit: CFI = 0.98, NNFI = 0.97, RMSEA = 0.075 (90% CI: 0.067 to 0.084; see Table 3). PD significantly predicted Observe (B = 0.09, R^2^ = 0.06, *p* = 0.008), indicating that participants diagnosed with PD exhibited greater levels of observing than non-PD participants. Factor loadings were all positive and significant. Inspection of modification indices revealed that each were small and below the recommended cut-offs (≥ 3.84). Thus, there was not substantial justification for pursuing MIMIC modeling to examine DIF.

### Anxiety sensitivity

To determine whether AS accounts for DIF in indicators of Observe, several MIMIC models were pursued. First, a measurement model with AS as a covariate was specified wherein the pathways of the AS variable predicting the item indicators were fixed to zero, yet the pathway to Observe was freely estimated. This model was associated with global fit indices that were good fit: CFI = 0.99, NNFI = 0.99, RMSEA = 0.076 (90% CI: 0.067 to 0.084; see Table 3). AS significantly predicted Observe (B = 0.02, R^2^ = 0.18, *p* < 0.001), indicating that as participants’ AS increased, level of observing increased as well. Again, the factor loadings were all positive and significant. Inspection of the modification indices revealed that item 1 (“When I’m walking, I deliberately notice the sensations of my body moving”), item 6 (“When I take a shower or bath, I stay alert to the sensations of water on my body”), item 26 (“I notice the smells and aromas of things”) and item 31 (“I notice visual elements in art or nature, such as colors, shapes, textures, or patterns of light and shadow”) were substantially above recommended cut-offs (≥ 3.84), with modification indices of 192.57, 237.55, 314.37, and 759.85, respectively.

Next, a MIMIC model was pursued in which the pathway between the AS covariate and these items (1, 6, 26, and 31) were freely estimated (see Fig. 1). The fully specified MIMIC model exhibited statistically significant better fit than the model with the covariate pathways fixed to zero, *χ*^2^_diff_ (4) = 109.47, *p* < 0.001). The chi-square statistic was significant (*χ*^2^ (36) = 66.43, *p* < 0.001) and the other indices indicated good global fit: CFI = 0.99, NNFI = 0.99, RMSEA = 0.035 (90% CI: 0.026 to 0.045). AS predicted higher levels of item 1 (B = 0.01, *p* < 0.001) and item 6 (B = 0.01, *p* < 0.001), and lower levels of item 26 (B= -0.01, *p* < 0.001) and item 31 (B= -0.01, *p* < 0.001). Factor loadings were all positive and significant. Inspection of the modification indices revealed no substantial areas of misspecification were substantively justified.

### Anxiety sensitivity Subscales

For the final step, the same MIMIC procedure was repeated, except this time the individual subscales of the ASI-3 were modeled as exogenous covariates (cognitive, social, and physical). The initial model included freely estimated pathways from the three covariates to Observe, whereas the pathways to the item indicators were fixed to zero. This model was associated with good fit. A correlation of *r* = 0.72, *p* < 0.001 was found between cognitive and physical subscales as well as between cognitive and social subscales (see Table 4). A correlation of *r* = 0.63, *p* < 0.001 was found between social and physical subscales (see Table 4).

**Table 4:** FFMQ and ASI Subscale Correlations

	FFMQ Observe	FFMQ Non-react	FFMQ Non-judge	FFMQ Describe	FFMQ Act with Awareness	ASI Physical		ASI Cognitive
FFMQ Non-react	0.33***							
FFMQ Non-judge	-0.58***	-0.13***						
FFMQ Describe	0.16***	0.41***	0.09***					
FFMQ Act with Awareness	-0.46***	-0.10***	0.55***	0.28***				
ASI Physical	0.28***	-0.08**	-0.29***	0.15***	-0.30***			
ASI Cognitive	0.33***	-0.14***	0.43***	-0.23***	-0.46***		0.72***	
ASI Social	0.29***	-0.14***	-0.41***	-0.22***	-0.36***		0.63***	0.72***

*Note.* ***p < 0.001

The chi-square statistic was significant (*χ*^2^ (41) = 208.36, *p* < 0.001; see Table 3), and the other indices indicated good global fit: CFI = 0.98, NNFI = 0.98, RMSEA = 0.052 (90% CI: 0.045 to 0.059; see Table 3). The pathway from physical AS to observe was positive, but not significant (physical AS -> observe, B = 0.01, *p* = 0.058). The other two freely estimated regression pathways were positive and significant (cognitive AS->observe, B = 0.04, *p* < 0.001; social AS ->observe, B = 0.01, *p* < 0.05). All the factor loadings were significant and positive.

The modification indices were inspected for the same items identified in the prior MIMIC model with the total AS score (items 1, 6, 26 and 31). The modification indices were large (i.e., exceeding a value of 10) for the pathways from the three covariates to items 1, 6, 26, and 31.

In light of these modification indices, a MIMIC model was specified such that all three subscales freely predicted the latent variable, as well as items 1, 6, 26, and 31.

The chi-square statistic was significant (*χ*^2^ (29) = 58.77, *p* < 0.01; see Table 3) and the other indices indicated good fit global fit: CFI = 0.99, NNFI = 0.99, RMSEA = 0.026 (90% CI: 0.016 to 0.036). The regression pathways of the cognitive subscale exhibited the same pattern of relations as the total ASI-3 scale. Physical AS (B = 0.008), cognitive AS (B = 0.02), and social AS (B = 0.009) were associated with higher levels of the observe latent variable (*p*’s < 0.05). Cognitive AS predicted higher levels of item 1 (B = 0.03, *p* < 0.001) and item 6 (B = 0.03, *p* < 0.001), and lower levels of item 26 (B= -0.02, *p* < 0.001) and item 31 (B= -0.03, *p* < 0.001). Social AS predicted higher levels of item 26 (B = 0.02, *p* < 0.05). The remaining pathways from the social and physical ASI-3 subscales to all items were not significant (*p* > 0.05). All factor loadings were significant and positive.

### Power analyses

Results of the post-hoc power analyses based on the RMSEA criteria indicated that all of the models investigated in this study evinced statistical power above 0.99, indicating adequate global power overall.

## Discussion

This study investigated the relationship between the FFMQ Observe facet items in a clinical sample of individuals with and without PD, with varying levels of AS. When PD was examined in relation to the Observe facet, PD significantly predicted observing, revealing that participants diagnosed with PD had greater levels of observing than those without PD. This is important to note clinically, as this finding suggests that those with PD are more likely to endorse observing more generally. PD did not appear to predict individual items of the Observe facet, thereby providing no evidence of DIF between internal or external items.

Those with higher AS were also found to observe more frequently. DIF was found in four items as a function of AS: two items related to internal focus (items 1 and 6) and two items related to external focus (items 26 and 31). This finding suggests that AS may influence how people observe internally and externally, regardless of diagnosis. Individuals with increased AS were found to rate these two items related to internal focus higher and two items related to external focus lower. This fits with prior findings that individuals who tend to scan their body for physical sensations likely spend more time worrying about and focusing on internal sensations [5]. Perhaps due to this type of focus, those with increased AS are also less likely to focus on the world around them. Although the proportion of variance accounted for in the DIF by the AS covariate was relatively modest, the significant relationships tell us an important story. Perhaps a reason the Observe facet of the FFMQ continues to show mixed results in the literature is because the facet does not differentiate between observing internal or external items. As a result, this facet may reveal mixed or inaccurate results of one’s observing tendencies.

When subscales of the ASI-3 were investigated, physical, cognitive and social AS were all associated with higher levels of observing. The physical subscale did not reveal DIF, which was contrary to our expectations. The social subscale predicted high levels of item 26, related to external focus (“I notice the smells and aromas of things”). There appears to be something about higher social AS that corresponds with an increased awareness of external smells. This finding warrants further exploration. The cognitive subscale showed the same pattern of results as total AS (individuals with increased AS were found to rate two items related to internal focus higher (items 1 and 6), and two items related to external focus lower (items 26 and item 31)). This was particularly interesting, as none of the items on the Observe facet that showed differences were cognitively related. Therefore, it appears there may be something about cognitive AS that is driving the influence of AS on internal vs. external indicators of observing. This makes sense in light of AS being described as a cognitive bias [18]. Additionally, individuals who worry about their thoughts may not be as aware when they focus on external stimuli. AS cognitive concerns often reflect catastrophic thinking, which may drive individuals to continue monitoring their interoceptive sensations, rather than external stimuli. Of note, the internal Observe items appear to be more tactile in nature (i.e. item 6: “When I take a shower or bath, I stay alert to the sensations of water on my body”). As anxiety often impacts what individuals observe, individuals who are more likely to catastrophize may interpret somatic symptoms as dangerous, leading to a vicious cycle of increased worry and narrower focus on physiological sensation [33].

### Limitations and Future Research

Several limitations exist in this study. First, diagnoses were self-reported based on previous practitioner diagnosis. Individuals were asked to answer yes or no to having received a diagnosis, and it is therefore possible this did not translate to the same diagnosis. For instance, individuals may have endorsed yes to major depressive disorder, however their provider assigned a diagnosis of a different mood disorder. As such, future research would benefit from clinicians conducting diagnostic evaluations of participants. Additionally, the results do not shed light on the directionality of observing internal stimuli and its impact on AS. As this study was cross-sectional in nature, future research may benefit from investigating this relationship further, perhaps through longitudinal research. Another limitation is that a priori power analysis was not run on this sample. However, given conventional standards for power analyses, the sample size exceeds most heuristic recommendations [13]. Post-hoc power analyses were also conducted.

A unique strength of this study is that it includes a fully Japanese sample. It is important to understand how cultural issues may or may not intersect with universal constructs, and we hope that our study can help be part of bridging this gap. As with all research conducted in a particular country, it is important to keep in mind that cultural factors may shape how respondents answered questions, and thus it may be less feasible to extrapolate findings from a Japanese sample to Western samples. While the FFMQ has been validated in a Japanese sample and has exhibited good psychometric properties [24], the ASI-3 was translated into Japanese for the first time. Therefore, it may be helpful for future studies to test its reliability and validity in Japanese. Future research would benefit from continuing to explore how culture or other identity factors play a role.

Importantly, several FFMQ subscales were negatively correlated with one another, which is consistent with previous findings obtained by Japanese samples [24]. In Japanese samples, those who score lower on the Observe facet tend to score higher on the Non-judge or Act with Awareness facets because the Observe facet appears to tap into a sensitivity to sensations and the Non-judge and Act with Awareness facets measure the ability to take attention away from cognitions or sensations. Negative correlations were found between the Non-react and Non-judge facets and the Non-react and Act with Awareness facet results are difficult to interpret as these correlations are very weak.

## Conclusions

This study utilized a transdiagnostic approach to understand differences in core components of anxiety related to observation. This study found that increased AS was associated with greater scores on observing internal items and lower scores on observing external items. It appears AS is important to study across clinical samples, to understand why those who have more internal focus perceive certain types of mindfulness as threatening. Future studies may benefit from a more thorough understanding and conceptualization of AS, across clinical and non-clinical samples, as anxious individuals may observe more internally or be more sensitive to their anxiety. As not all facets of mindfulness have been found to neatly correlate with reduced anxiety [9], it is important to continue to conduct transdiagnostic research to identify which types of mindfulness will be most beneficial for each person. Future research may benefit from expanding this work to a non-clinical sample, as observation is an important component of mindfulness that extends to all types of individuals.

Based on the findings from this study, it may be beneficial to develop a scale that more adequately measures internal and external observation, including cognitive and emotional observation. As the field is beginning to shift away from a nomothetic approach of treating specific diagnoses and towards an idiographic approach of better understanding symptoms at the individual level, we hope to continue to understand the function of types of observation which may lead us to the ability to implement more accurate personalized treatment.

## Electronic supplementary material

Below is the link to the electronic supplementary material.

Supplementary Material 1

## Data Availability

The datasets used and/or analyzed during the current study are available from the corresponding author on reasonable request.
