# Supplementary material for: Influence of anxiety sensitivity on the observe facet of the five facet mindfulness questionnaire: differential item functioning in a clinical population
Source: BMC Psychiatry. 2025 Apr 1;25:312. doi: 10.1186/s12888-025-06488-x (PMC11959827; doi:10.1186/s12888-025-06488-x)
Supplement: Supplementary file 1 — Supplementary Material 1 [file 12888_2025_6488_MOESM1_ESM.docx]

**Supplemental Methods and Results**

**Method**

The data analytic strategy was largely identical to that pursued in the principal body of the paper with the exception that Major Depressive Disorder (MDD) was considered as a binary covariate (i.e., presence or absence) in the MIMIC model as opposed to Panic Disorder (PD).

**Result**

*Major Depressive Disorder*

A MIMIC model was pursued to determine whether MDD accounts for DIF in indicators of the latent variable of Observe. Akin to the model presented for PD, a measurement model with MDD as a covariate was specified wherein the pathways of MDD predicting the item indicators were fixed to zero, yet the pathway to Observe was freely estimated. This model was associated with global fit indices that were between adequate to good fit: CFI =0.91, NNFI =0.88, RMSEA =0.08 (90% CI: 0.072 to 0.088). PD significantly predicted Observe (B =0.14, R^2^ =0.01, *p*<0.001), indicating that participants diagnosed with PD exhibited greater levels of observing than non-PD participants. Factor loadings were all positive and significant, with unstandardized coefficients from 0.84 to 1.34. Inspection of the modification indices revealed that item 1 (“When I’m walking, I deliberately notice the sensations of my body moving”) and item 31 (“I notice visual elements in art or nature, such as colors, shapes, textures, or patterns of light and shadow”) were substantially above recommended cut-offs (≥3.84), with modification indices of 20.21 and 12.63, respectively.

The fully specified MIMIC model exhibited statistically significant better fit than the model with the covariate pathways fixed to zero, *χ*^2^_diff_ (2) =30.97, *p*<0.001). Although the chi-square statistic was significant (*χ*^2^ (25) =258.72, *p*<0.001), the other indices indicated reasonable to good global fit: CFI =0.92, NNFI =0.89, RMSEA =0.078 (90% CI: 0.070 to 0.087). MDD predicted higher levels of item 1 (B =0.24, R^2^ =0.322, *p*<0.01) and lower levels of item 31 (B= 0.85, R^2^ =0.280, *p*<0.001). Inspection of the modification indices, in conjunction with considering theoretical rationale, revealed no further justification for pursuing additional MIMIC models.
